# A multi-chamber tissue culture device for load-dependent parallel evaluation of tendon explants

**DOI:** 10.1186/s12891-019-2896-2

**Published:** 2019-11-18

**Authors:** Endre Soreide, Janet M. Denbeigh, Eric A. Lewallen, Roman Thaler, Rebekah M. Samsonraj, Dakota L. Jones, Wei Xu, Dirk Larson, Lars Nordsletten, Sanjeev Kakar, Andre J. van Wijnen

**Affiliations:** 1 Department of Orthopedic Surgery, Mayo Clinic, 200 First Street SW, Rochester, MN 55905 USA; 2 Division of Orthopedic Surgery, Oslo University Hospital, Kirkeveien 166, 0424 Oslo, Norway; 3 Faculty of Medicine, University of Oslo, Oslo, Norway; 4 Department of Biological Sciences, Hampton University, Hampton, VA 23668 USA; 5 Department of Physiology and Biomedical Engineering, Mayo Clinic, 200 First Street SW, Rochester, MN 55905 USA; 6 Department of Orthopedics, Second Affiliated Hospital of Soochow University, 1055 Sanxiang Road, Suzhou, 215004 China; 7 Department of Biomedical Statistics and Informatics, Mayo Clinic, 200 First Street SW, Rochester, MN 55905 USA; 8 Department of Biochemistry and Molecular Biology, Mayo Clinic, 200 First Street SW, Rochester, MN 55905 USA

**Keywords:** Explant culture, Tendon, Ligament, Ex vivo, Musculoskeletal tissue, Tissue engineering

## Abstract

**Background:**

Injuries in the musculoskeletal system, such as tendon and ligament ruptures, are challenging to manage and often require surgical reconstructions with limited long-term success. Thus, characterizations of these tissues are urgently needed to better understand cellular mechanisms that regulate tissue homeostasis and healing. Explant culturing systems allow for ex vivo analysis of tissues in an environment that mimics the native microenvironment in vivo.

**Methods:**

Collaborative efforts within our institution facilitated the establishment of a novel explant culturing system. Tissue specimens cultured in single wells, with individual applied loading and/or biological environment, allowed characterization of tissue cultured under a variety of biological loading conditions. Quantitative PCR analysis for selected gene markers was our primary outcome.

**Results:**

Data were stratified for analysis by either culture environment or loading condition. Our gene expression results show that specimens clustered by culture condition may differ in molecular markers related to ECM production (e.g., Col1a1, Adamts4) and/or organization (e.g., Tnc, Dnc). In contrast, loading condition did significantly alter the median gene expression levels of tissues in comparison to unloaded control samples, although gene expression values related to ECM degradation (e.g., Mmp1, Mmp10) were altered in tendons cultured under tension in the device.

**Conclusion:**

Our study demonstrates promising utility of a novel explant culturing system for further characterization of musculoskeletal tissues such as native tendons and ligaments, as well as pathologic fibrotic tissues resulting from arthrofibrosis or Dupuytren’s disease.

## Background

Injuries to connective tissues within the musculoskeletal system (e.g., tendon and ligament) are common among physically active people [1–3]. Due to limited healing potential for ligament injuries, they are challenging to manage, and surgical reconstruction is often required to restore the stability and function of the affected joint [4, 5]. Although surgical techniques used in ligament reconstructions are advancing, long-term clinical data have demonstrated persistent and recurrent joint-related symptoms and instability after reconstruction [6, 7]. Most of these surgeries involve use of tendon autografts. As such, previous observations of ACL reconstructions highlight the insufficiency of current surgical repair methods to restore and preserve long-term function [2, 8]. Therefore, studies characterizing both native tendons and healing tendon grafts will improve our understanding of endogenous cellular processes that normally maintain homeostasis and provide guidance toward revealing strategies that improve long-term patient outcomes.

An ideal ligament reconstruction has full functional integration between the soft tissues and adjacent bones in order to withstand physical strains of the intra-articular environment and restore joint stability. Ligament injuries are commonly reconstructed using tendon grafts inside bone tunnels [1, 9], although tendon-to-bone tunnel healing can sometimes be delayed or inadequate [10]. Additionally, tendons fundamentally differ from ligaments in morphological composition, extracellular matrix content, architecture, and biomechanical properties [11, 12]. Further, remodeling of connective tissue, including tendons and ligaments, involves altering the content and/or architecture of the extracellular matrix (ECM). Thus, enhancing ECM deposition may improve the biomechanical characteristics of grafted tendons and allow better functional outcomes of ligament reconstructions.

Explant tissue culture provides the opportunity to study cells in a natural three-dimensional ECM, thereby mimicking an important physical component of the in vivo microenvironment [13–15]. As the cellular homogeneity and contacts are preserved, intercellular signaling and communication are maintained, allowing investigation of other parameters important for regulation of homeostasis and healing. Mechanical loading, for example, plays a critical role in maintaining tissue homeostasis in native musculoskeletal tissues [16] which is believed to act via strain-induced signaling of mechanotransduction pathways [17]. A variety of stress levels have been shown to induce anabolic response in ligament and tendon cells [17, 18], while stress deprivation is correspondingly associated with a decline in mechanical properties [16] and matrix degradation due to matrix metalloproteinase expression [19]. The present study therefore establishes a novel explant culturing system that allows experimental isolation of the cellular effects from biomechanical forces applied to tendon tissues.

## Methods

An innovative explant culturing system was established in close collaboration with the Department of Engineering at Mayo Clinic (Rochester, MN). An important criterion for the culturing system was to facilitate individual loading of the tissue specimens, and controlled manipulation of biological parameters (e.g., growth factors, different media, and inhibitory reagents). Tissue specimens were cultured in single independent wells, allowing multiple setups regarding environmental condition, and biomechanical loading conditions in culture. Thus, allowing comparison analysis of specific loads and specific enrichment concentrations to define the effect of load versus various growth media factors on extra cellular matrix synthesis, cellular composition and organization, and tissue architecture for defining the biomechanical properties of an explant tissue.

### Tissue handling

**Fig. 1:**
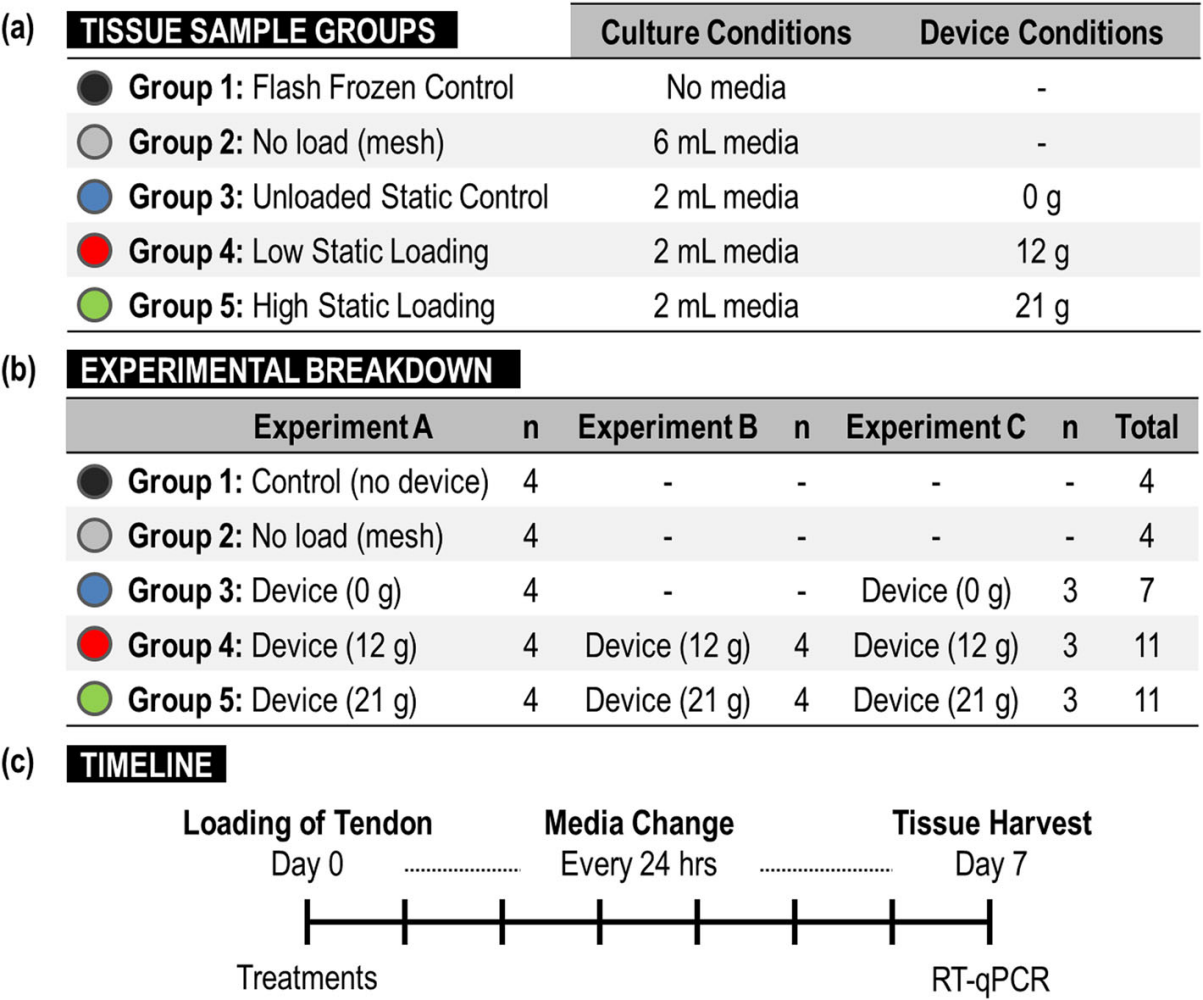
(**a**) Breakdown of culture and device experimental conditions designed to isolate the effects of culture and loading conditions, (**b**) tissue sample groups, and (**c**) timeline of experiments

Semitendinosus tendons from New Zealand white rabbits (mean + sd weight of 3.62 + 0.27 kg) were donated by Mayo Clinic researchers after the conclusion of their studies that were independently approved by the Institutional Animal Care and Use Committee (IACUC). Tissues were harvested immediately following sacrifice to ensure consistency and maximize similarity to an in vivo setting. Tendons, weighing 84.7 + 23.9 mg, were excised proximally at the tendon-muscle junction site and distally at the tibia insertion site under aseptic conditions. Any remaining muscle or other soft tissue was carefully removed prior to immersion in transport media comprised of advanced Modification of Eagle’s Media (aMEM) (Invitrogen, Carlsbad, CA), supplemented with 10% fetal bovine serum (FBS), 1% penicillin/streptomycin, and L-glutamine (Invitrogen, Carlsbad, CA), standardized to a pH of 7.2. All tissues were divided into five experimental groups: (1) Control samples snap frozen immediately after collection, (2) tissues cultured in a 60 mm dish w/mesh, (3) tissues cultured in the explant device without tension, (4) with ~ 12 g of tension, and (5) with ~ 21 g of tension applied to the tissues using a suture system (Fig. 1).

### Explant tension culturing system

**Fig. 2:**
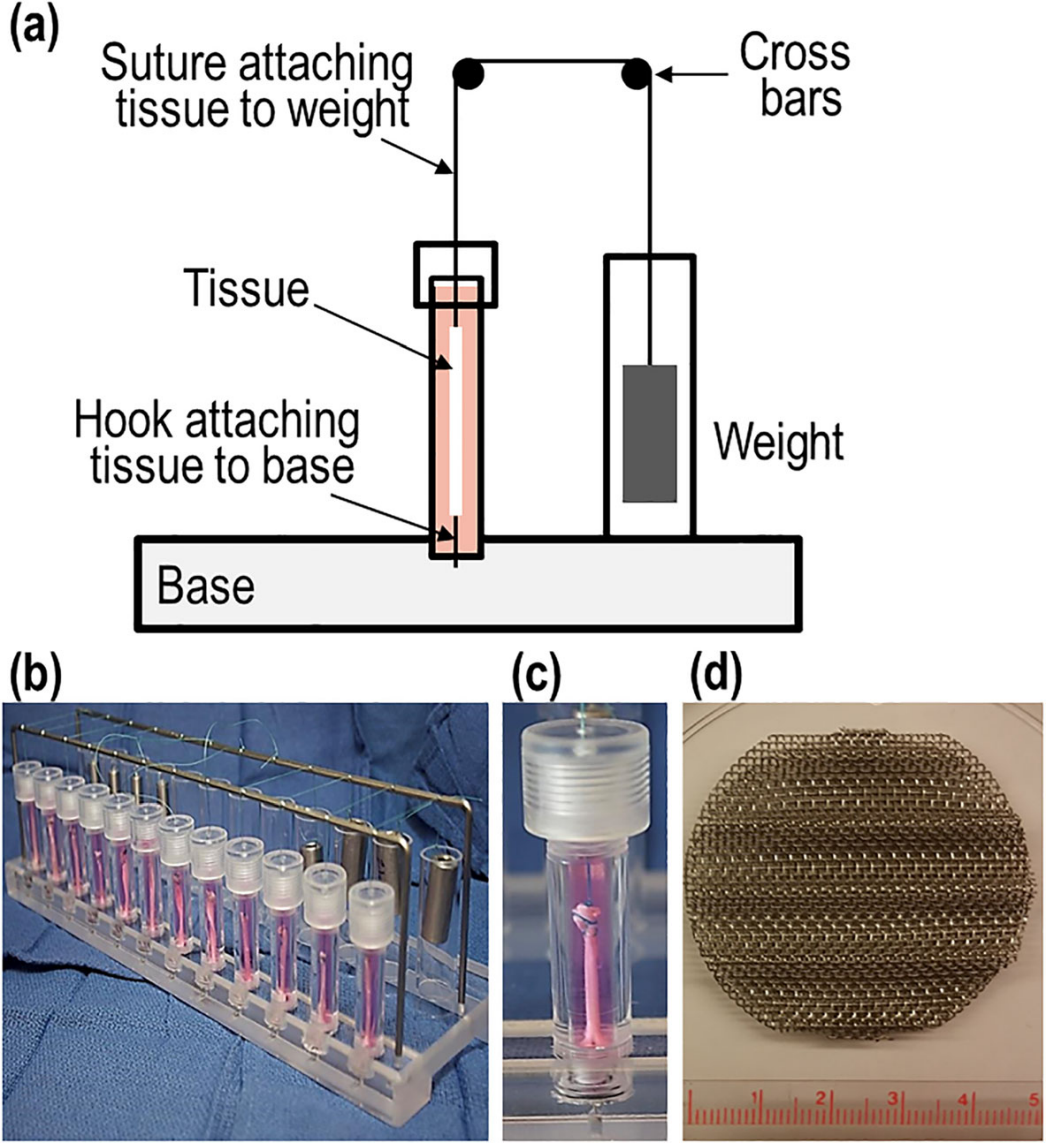
(a) A lateral view diagram of the tension device components depicting a chosen weight applying tension to the tissue via a suture. (**b**) A photographic image of the wells arranged in parallel to allow side-by-side experimental manipulations. (**c**) A close-up view of one tissue culture well containing a rabbit semitendinosus tendon secured to the device by suture and base hook. (**d**) A close-up view of the custom-made mesh for tendon culture. Scale in cm

Cylindrical wells were fabricated from Poly (methyl methacrylate) (PMMA) rods (5 × 1 cm) by removing a central core of 5 mm in diameter. Both ends of the cylinders were threaded to allow attachment of cylinders to the base and top cap. Two-millimeter central openings were added to cylinder caps to ensure sufficient gas exchange and facilitate passage of a holding suture. The bottom between the base and the cylindrical well was sealed with a rubber washer to prevent leakage. A stainless steel hook securely fixed with a screw was placed to the bottom of each well. A suture was then placed between the tissue specimen and a specified load was guided over two crossbars attached to the device base. Twelve independent cylindrical tissue culture wells were fixed to the base of the device (Fig. 2). All parts of the device were sterilized prior to conducting the experiments by Ethylene Oxide (EtO) gas to prevent contamination without causing damage to the PMMA.

### Tissue culture conditions

Following removal of transport media and thorough rinsing of the tendons in phosphate buffered saline (PBS), an Ethibond 3–0 (Ethicon, Sommerville, NJ, USA) holding suture was added to the proximal end, under aseptic conditions. The distal end of the tissue specimen was then attached to the stainless steel hook in each well bottom before the cylinder was passed over the graft and securely attached to the device base. The suture was passed through the opening in the top of the cap and a load was applied (or not applied in the case of control samples). To assess correlation between applied tension to the tissue and gene expression, two different loads (~ 12 g and ~ 21 g), in addition to unloaded (0 g) control specimens, were compared. Applied loads were equivalent to 0.12 Newtons (N), 0.21 N and 0 N, respectively. Even though the applied loads were multiple times the weight of the tendon tissues itself, our main intention was to maintain the tissue under tension in culture while avoiding micro damage to the sub-synovial connective tissue, including microtears and rupture of thin fibrils. According to Morizaki et al. [20], the threshold of applied force for damage to rabbit tendons appears to be ~ 200 mN. Similar tensions (0.04 N – 0.2 N) were applied by Arnoczky et al. [17] when assessing the response of rat tail tendons to static tensile loads. We therefore set our maximum load to 0.21 N, with a group receiving half that load (0.12 N) and a third group receiving no load (0 N). None of the tissue samples were pre-tensioned. Growth media was added to each well in the following formulation: 900 μL of aMEM, 5% platelet lysate, 1% penicillin/streptomycin, and L-glutamine.

Tissue specimens for the external control group were cultured in 60 mm dishes, placed in a custom-made mesh to keep tendons straight, but without any longitudinal or compressive forces applied to the tissue. Tissues were maintained in 5 mL per dish of the same 5% platelet lysate media. The explant culturing system and dishes were placed in a controlled incubator maintained at 37.5 °C, 95% humidity and 5% CO_2_. Media were changed every 24 h. All tissues in group 1 (day 0) were harvested from animals immediately after euthanasia, debrided of non-tendon tissue, rinsed in PBS, and snap frozen in liquid nitrogen. For specimens in treatment groups 2 thru 5, harvested tissues were rinsed in PBS, debrided of non-tendon tissue, cultured for seven days, rinsed in PBS, and snap frozen in liquid nitrogen for further analysis by RNA extraction and real time quantitative PCR (RT-qPCR).

### RNA isolation and mRNA expression analysis

RNA was isolated using the miRNeasy kit (Qiagen, Hilden, Germany). Isolated RNA was reverse transcribed into cDNA using the SuperScript III first strand synthesis system (Invitrogen, Thermo Fisher Scientific, Waltham, MA, USA). Gene expression for selected gene markers was quantified using RT-qPCR whereby each reaction was performed with 2.5 ng of cDNA per 10 ul, QuantiTect SYBR Green PCR kit (Qiagen, Hilden, Germany), and the CFX384 real time system machine (Bio-Rad, Hercules, California, USA). Transcript levels were quantified using the 2^ΔΔCt^ method and normalized to the housekeeping gene Actb (set at 100).

### Statistics

Heat maps of the RT-qPCR data for (i) culture conditions and (ii) device conditions were generated using GENE-E v.3.0.215. Analysis of variance (ANOVA) was used to test for significant differences between (i) the three culture conditions or (ii) the three device conditions using SAS v.9.4 (SAS Institute Inc., Cary, NC). To account for multiple comparisons, we adjusted the false discovery rate (FDR) using the Benjamini-Hochberg procedure. Box and whisker plots identify the median and interquartile range of the data sets, while outliers were plotted as individual points. In all cases, significance was defined as *p* < 0.05. Venn diagrams made within FunRich v3 [21] were used to show the distribution of genes with increasing or decreasing fold changes for the mesh dish tendons and the 0 g tendons relative to frozen controls. The areas of each circle were proportional to the number of genes in each group.

## Results

### Tissue handling

A total of 37 fresh frozen tendons were collected from rabbits donated to our study and divided into five groups (1 thru 5) and three experiments (A, B, and C) (Figs. 1, 2). Tissues in groups 1 and 2 were not cultured in the device, but served as unloaded controls cultured independent of our novel tensioning system. We did not observe any errors or complications related to the use of the culturing system for explant tendons in groups 3, 4, or 5.

### Unloaded explant tendon culture conditions

**Fig. 3:**
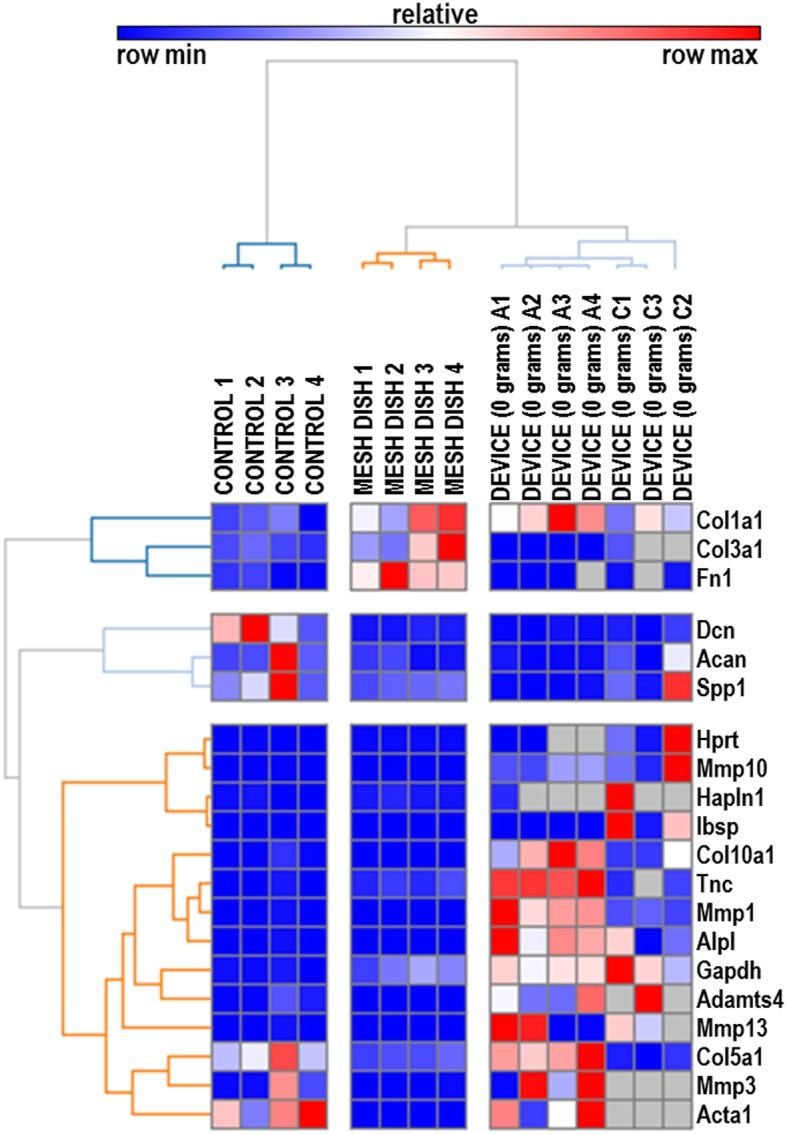
RT-qPCR data from samples cultured in unloaded conditions. Gene-Mania analysis depicting clear clustering of gene expression data by unloaded culture condition (control, mesh dish, or device with 0 g)

**Fig. 4:**
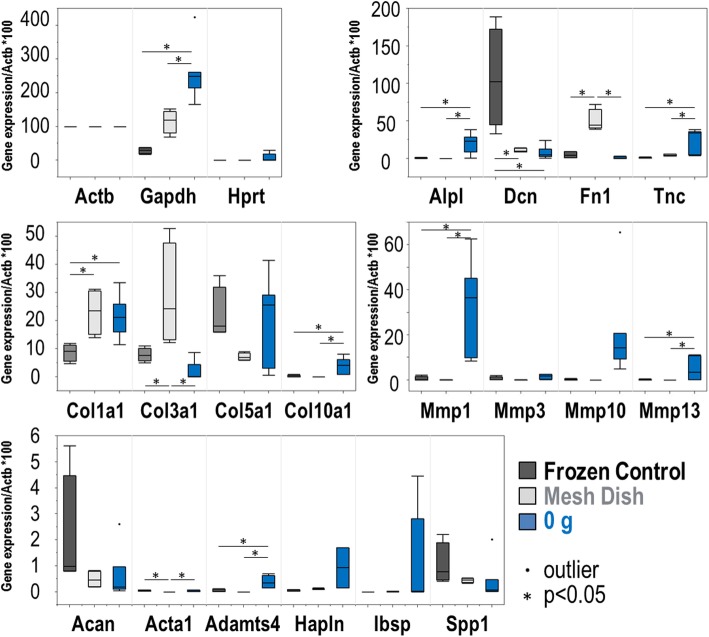
Box and whisker plots of gene expression levels (obtained by RT-qPCR) that allow comparison of samples collected from different culture conditions

A set of 20 rabbit-specific qPCR primer pairs (see Additional file 1 for a complete list) were used to characterize biological effects of different culture conditions (substrate type, or volume of media). Analysis of these data by hierarchical clustering revealed clear patterns of cladistic grouping among control samples, samples cultured in a custom mesh sandwich in dishes, and samples cultured in the device without weight. Interestingly, Col1a1, Col3a1, and Fn1 markers were elevated in both the mesh dish- and device- cultured tissues, whereas Dcn, Spp1, Col5a1, and Acta1 were upregulated in control tissues (Fig. 3). Further, compared to control samples, gene expression of Col1a1 was significantly increased in both mesh dish and unloaded samples. While mesh samples demonstrated significantly increased expression of Col3a1, 0 g samples had increased expression of Col10a1. Mmp1 and Mmp13 expression was significantly increased in 0 g samples (Fig. 4).

**Fig. 5:**
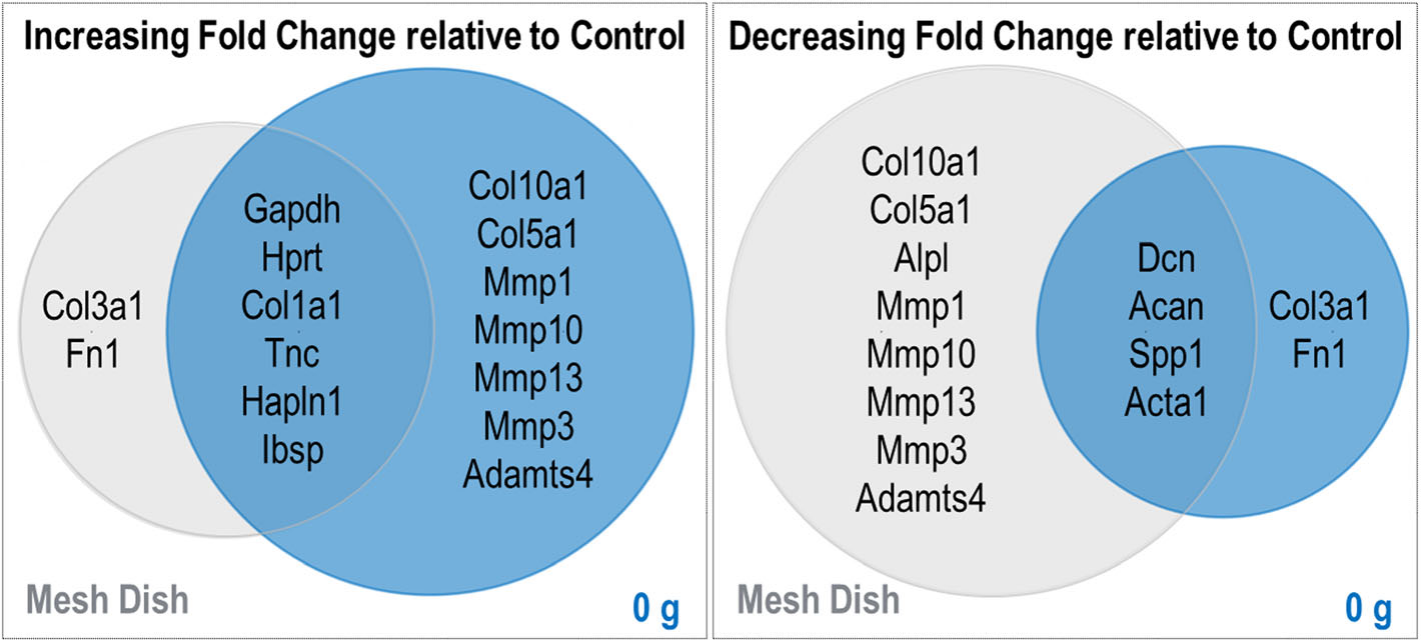
Venn diagrams showing median fold change comparisons among treatments that differed only by culture condition as compared to flash frozen control tissue

Overall, adjusted FDR values for collagen (Col1a1, Col10a1, Col3a1) and matrix metalloproteinase markers (Mmp1, Mmp13) were significantly different among the three culture condition groups. The group differences for Mmp10 data were nearly significant (*p* < 0.053; Table 1). Additionally, Fn1 and Alpl values were statistically different among the groups (Table 1). Further, the noted findings were confirmed by the FunRich diagram, demonstrating an increased fold-change for both Col10a1 and Col5a1 and matrix metalloproteinase markers (Mmp1, Mmp3, Mmp10 and Mmp13) in the 0 g samples compared to the flash frozen controls (Fig. 5).

**Table 1:** *P*-values comparing unloaded culture conditions to flash frozen tendon samples.

Adjusted (FDR)				
GeneID	Overall	Mesh vs Control	Mesh vs Weight - 0 g	Control vs Weight - 0 g
Gapdh	**0.0001**	***0.0616***	**0.0030**	**< 0.0001**
Hprt	0.3417	**–**	**–**	**–**
Col1a1	**0.0163**	**0.0156**	0.6945	**0.0156**
Col10a1	**0.0068**	0.8593	**0.0125**	**0.0125**
Col3a1	**0.0099**	**0.0294**	**0.0108**	0.4104
Col5a1	0.2222	**–**	**–**	**–**
Tnc	**0.009**	0.6462	**0.0200**	**0.0159**
Dcn	**0.0012**	**0.0024**	0.8955	**0.0015**
Fn1	**< 0.0001**	**< 0.0001**	**< 0.0001**	0.5505
Acan	0.1984	**–**	**–**	–
Hapln1	0.0910	0.8492	0.0815	0.0815
Alpl	**0.0035**	0.9496	**0.0062**	**0.0062**
Ibsp	0.3069	**–**	**–**	**–**
Spp1	0.2975	**–**	**–**	**–**
Mmp1	**0.0065**	0.9589	**0.0105**	**0.0105**
Mmp10	***0.0525***	0.9834	***0.0642***	***0.0642***
Mmp13	**0.0334**	0.9581	**0.0416**	**0.0416**
Mmp3	0.1048	**–**	**–**	**–**
Acta1	**0.0230**	**0.0258**	**0.0258**	0.9055
Adamts4	**0.0084**	0.7149	**0.0146**	**0.0146**

Bold text indicate all comparisons that yielded significant (*p* < 0.05) or marginally significant (*p* < 0.06, italicized) differences between experimental groups. *FDR* False discovery rate

### Comparison of different loading strategies in Tendon Culture Device

**Fig. 6:**
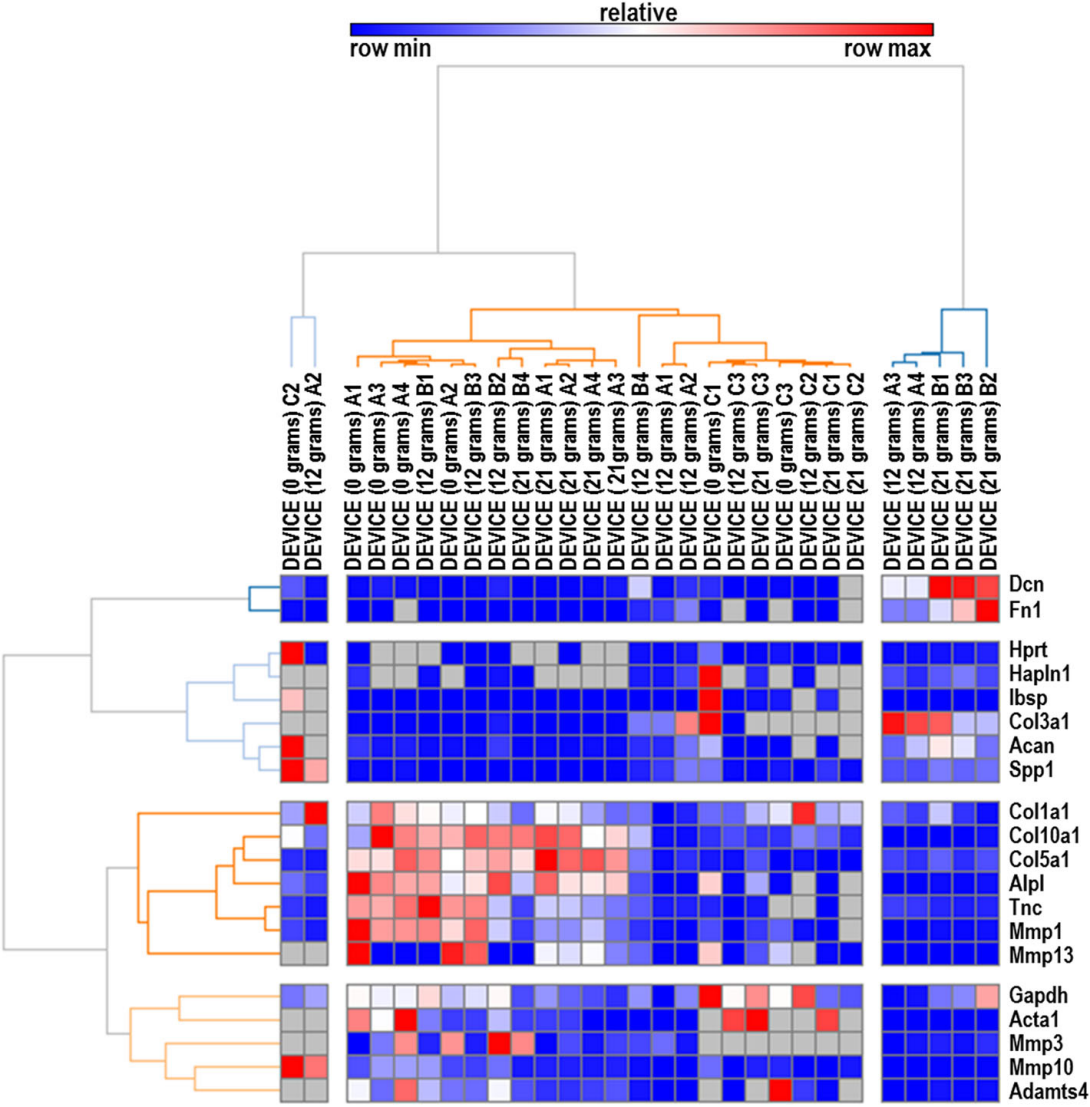
RT-qPCR results comparing loaded culture conditions using Gene-Mania for clustering

**Fig. 7:**
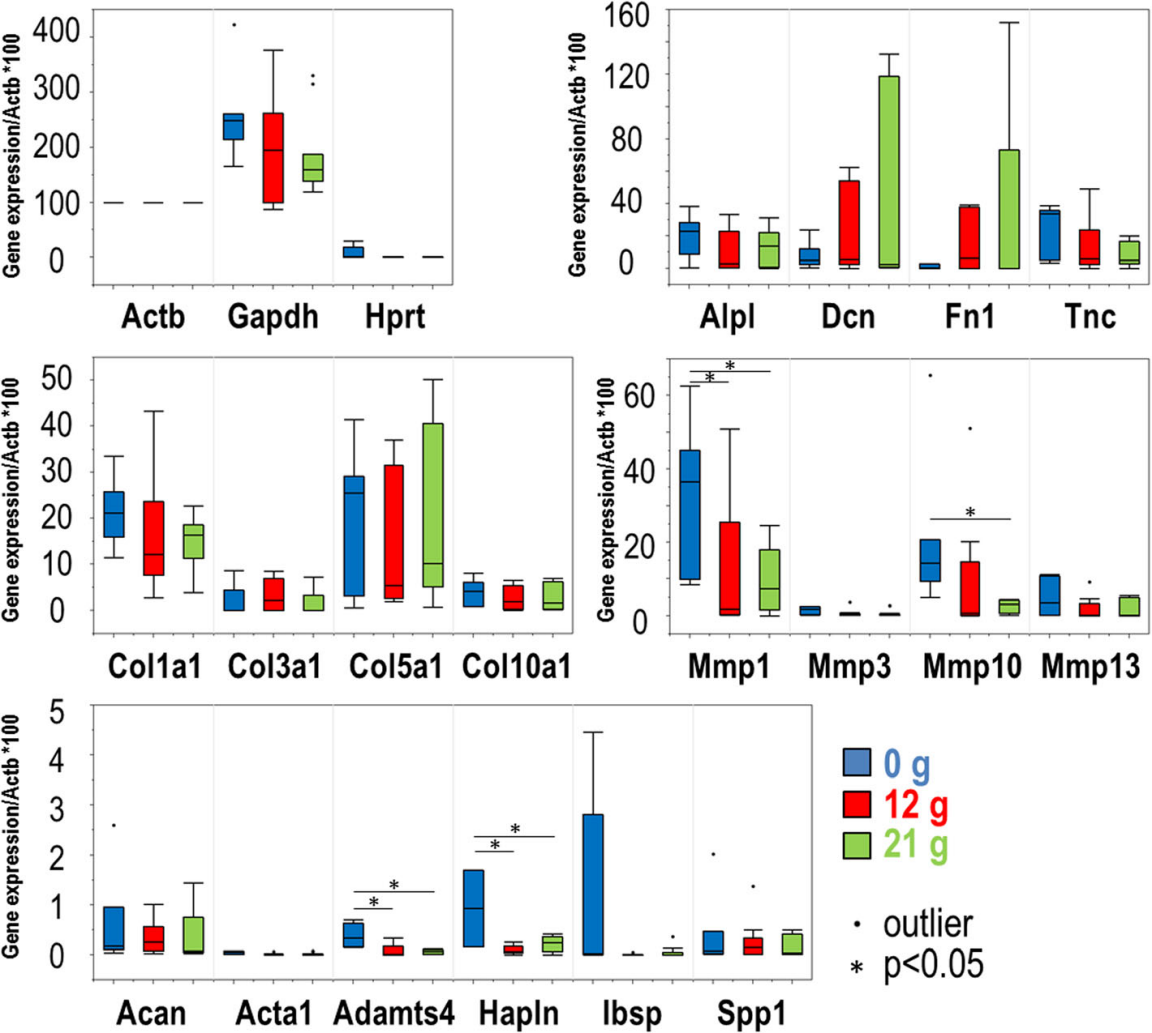
Box and whisker plots of gene expression levels (obtained by RT-qPCR) that allow comparison of samples collected from different device conditions

**Fig. 8:**
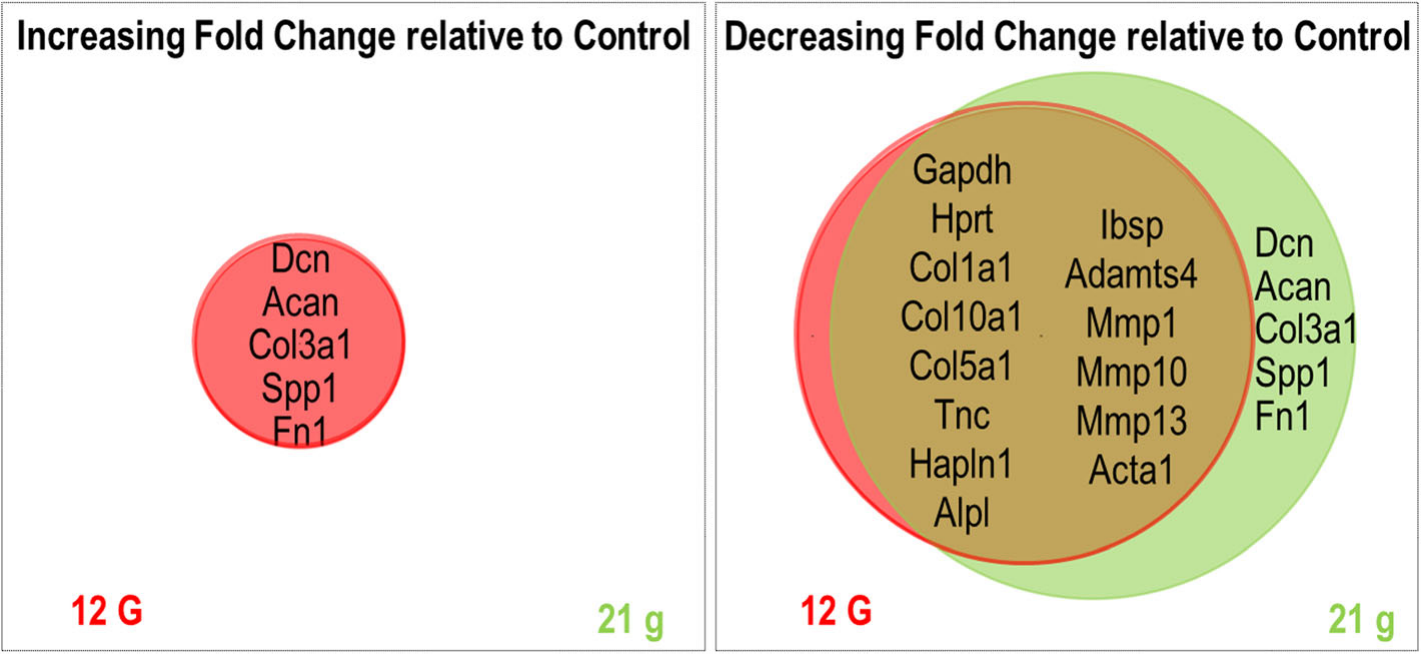
Venn diagrams comparing changes in gene expression for the device tension conditions, with fold changes for 12 g and 21 g weights relative to 0 g

Hierarchical clustering analysis did not reveal a clear pattern of distinction between samples cultured in the device (with or without tension). Of note, however was the clustering of molecular markers into distinct clades formed primarily by (i) an Mmp and Collagen cluster, (ii) a cluster of mineral deposition proteins, and (iii) a cluster comprised of Dcn and Fn1 (Fig. 6). There was no difference in expression between zero loading and the loaded samples for the various collagen makers. We did note a significant increase in Mmp1 expression in 0 g load compared to both 12 g and 21 g. In addition, Mmp10 was increased in 0 g tendons, however the relationship was significant only when compared to 21 g samples. Zero load samples also demonstrated increased expression of Adamts4 and Hapln1 (Fig. 7). Importantly, differences among device-cultured samples for the following primer sets were significant (*p* < 0.05): Hapln1, Mmp1, Mmp10, and Adamts4. Nearly significant (*p* < 0.06) differences identified Ibsp and Mmp13 as interesting molecular markers related to the biological response of tendon tissues exposed to different levels of strain (Table 2). Increasing and decreasing fold changes (relative to 0 g controls) are represented by Venn diagrams in Fig. 8.

**Table 2:** *P*-values comparing device tension conditions.

Adjusted (FDR)				
GeneID	Overall	0 g vs. 12 g	0 g vs. 21 g	12 g vs. 21 g
Gapdh	0.1742	–	–	–
Hprt	0.0971	0.0992	0.0992	0.9260
Col1a1	0.4201	–	–	–
Col10a1	0.5014	–	–	–
Col3a1	0.6727	–	–	–
Col5a1	0.5088	–	–	–
Tnc	0.0868	0.1742	0.0882	0.4345
Dnc	0.2713	–	–	–
Fn1	0.3102	–	–	–
Acan	0.6376	–	–	–
Hapln1	**0.0190**	**0.0171**	**0.0300**	0.5276
Alpl	0.2062	–	–	–
Ibsp	***0.0576***	***0.0528***	***0.0528***	0.9203
Spp1	0.5607	–	–	–
Mmp1	**0.0357**	**0.0462**	**0.0450**	0.6833
Mmp10	**0.0385**	0.1277	**0.0348**	0.3035
Mmp13	***0.0554***	***0.0611***	***0.0611***	0.6988
Mmp3	0.3695	–	–	–
Acta1	0.1329	–	–	–
Adamts4	**0.0008**	**0.0012**	**0.0012**	0.6580

Bold text indicates all comparisons that yielded significant (*p* < 0.05) or marginally significant (*p* < 0.06, italicized) differences between experimental groups. *FDR* False discovery rate

## Discussion

The novel explant culturing system described herein is a useful tool for ex vivo studies of tissues (and grafts) that need to withstand tensile forces. The appliance allows assessment of natural intrinsic mechanisms and effects of the in vivo biological microenvironment, with particular focus on musculoskeletal tissues (e.g., tendon, ligament, Dupuytren’s disease-afflicted palmar fascia, and arthrofibrotic knee joint capsule tissues).

Mechanical loading is an important contributor to both the healing and homeostasis of connective tissues, particularly tendons and ligaments [22]. Our ex vivo culturing system attempts to mimic the in vivo loading environment promoting native cell ECM reconstruction and maintenance, which correlates to the constant baseline tension applied to tendons and ligaments. Intercellular gap junctions, intercellular actin cytoskeleton, cell surface receptors and signaling molecules allow for modification of the ECM by altered gene expression via mechanotransduction. Thus, the presented culturing device enables comparison of different loading conditions to increase the validity, and potential clinical applicability of future gene expression analyses of tendon explants.

A panel of biomarkers were carefully selected a priori to allow assessment of gene expression within the tissue while in culture. In particular, we selected biomarkers related to ECM production and remodeling, in addition to inflammatory signals that may affect the production,organization and degradation of the ECM [23]. Future mechanistic evaluations of genes controlling deposition and spatial organization of the ECM will improve our understanding of early onset pathological conditions, including tendinopathies. In addition, such studies will reveal important target genes for biological enhancement to treat pathological conditions, as well as to improve the restorative capabilities of musculoskeletal tissues.

Our comparison of culturing conditions revealed increased gene expression of Col1a1, the most important contributor to mechanical strength in tendon tissue. In addition, Col3a1, known to mature into Col1a1, was significantly upregulated. Combined with a significant increased expression of Fn1, an ECM protein ensuring connection between fibroblasts and collagen fibrils, a notable remodeling process was detected in tendons cultured in our device. Further, we noted increased collagenase expression in the unloaded samples compared to the samples cultured under loaded conditions with increased strain on the tissue. A similar pattern of increased collagenase expression in response to unloading has been shown previously in tendons [24]. We also observed an increase in the expression of Mmps in our 0 g (0 N) tissue compared to those under tension, which agrees with existing literature, and suggests that a lack of graft tension leads to matrix degradation [17–19]. This finding reinforces the importance of mechanical loading for tissue homeostasis [22], and the need to apply tensile forces to explant cultures during the ex vivo study of tendons and ligaments for valid outcome data.

We observed a difference in gene expression in loaded tendon tissues compared to our control, snap frozen tendon tissues. These differences reflect microenvironmental culture exposure differences and are likely to affect the tendon phenotype by modes described for cartilage explant cultures [25]. Thus, the included control was crucial to demonstrate the magnitude and nature of difference as a result of the exposure. However, future studies will be made between cultured tissues maintained in parallel chambers simultaneously to account for sample variability. The variability in gene expression between cultured samples is likely due to regional differences within the tendon tissue and/or asymmetric, asynchronous necrosis patterns. Varying regional levels of nutrients and oxygen within the tissue may lead to heterogeneous necrosis. A future modification of the culturing device could be an improved nutrient and gas exchange system to better standardize fine-scale necrosis and reduce gene expression variability.

Further, characterization of ligament and tendon tissue is important for possible enhancement of ligament reconstructions and the involved biological transformation of tendon graft into tissue with native ligament-like properties, referred to as “ligamentization” [12]. Affecting the gene expression by biological enhancement of the controlling cell signaling pathways may favorably alter the deposition, content and organization of the ECM to provide better outcomes of ligament reconstructions due to improved biomechanical capabilities of the grafted tissue to withstand both short- and long-term tensile demands.

Mechanical loading is crucial for tendon development, homeostasis and repair [22]. Cellular spatial orientation is influenced by the direction of load in explant mechanical loading systems [26]. In addition, application of mechanical load has shown to promote tendon-like tissue with enhanced biomechanical properties [27, 28]. Thus, morphological organization defining the biomechanical properties may be altered in explant systems. Other single-chamber tissue tension explant culturing systems have been published [13, 24, 29, 30]. Our setup, using a constant strain is similar to previously described systems to show that application of strain to tissue in culture may reduce collagen degradation of the ECM [30]. The main advantage of our explant tension culturing system is the single tissue sample in single well concept. This enables comparisons of various drug, growth factor or morphogen concentrations to be done simultaneously. In addition, different mechanical loading conditions can be assessed in parallel, to quantify the importance of mechanical load compared to enrichment at specific concentrations. In sum, this facilitates tissue engineering tendon constructs under multiple experimental conditions [31]. Our system is also specifically designed to be resource conservative such that small aliquots of costly cell culture medium supplements can be added without altering the treatment regimen. Importantly, the separate wells in our system will reduce (or eliminate) cross-well contaminations.

We found increased gene expression of specific gene markers associated with ECM production and organization, indicating that the explant device provides a favorable culture environment. Also, we did not observe a rise in inflammation response. However, the static loading condition used in the presented explant culture device does not match a in vivo physiological loading condition for tendons or ligaments. Hence, a dynamic loading capability is a further improvement of the culturing device that we are currently addressing to better mimic both physiologic and pathologic conditions with cyclic loading of the musculoskeletal tissue.

Tendon or ligament cells isolated from harvested tissue and cultured in three dimensional gels, scaffolds or composites have been used for assessing the effects of biological environments and loading conditions on gene expression [32–38]. Increased gene expression of Col1a1 and Col3a1 were achieved in vitro after two weeks of mechanical stimulation of stem cell-collagen sponge constructs [36]. In contrast, the presented culturing device enables characterization of the cells kept within the native ECM, which may increase data validity and translation of our results into clinical settings. Further, preserving the native extracellular environment is useful for more specific characterization, and constituting intervention studies of pathologic musculoskeletal tissues (e.g., fibrotic palmar fascia in Dupuytren’s disease and arthrofibrotic knee joint capsule tissue). Increased knowledge of the involved signaling pathways in healthy ligament tissues will facilitate evolution of novel regenerative repair strategies to replace the current gold standard ligament reconstructions via tendon graft. In contrast to a ligament reconstruction, a ligament repair would advantageously preserve the proprioception and native transition zones between ligaments and bones, optimizing the potential for enhanced long-term functional patient outcomes.

## Conclusion

Our study demonstrates promising utility of a novel multi-chamber explant tissue culturing system, enabling variable mechanical loading conditions for further characterization of musculoskeletal tissues such as native tendons and ligaments, as well as pathologic fibrotic tissues resulting from arthrofibrosis and Dupuytren’s disease.

## Supplementary information

**Additional file 1:** RT-qPCR primers for gene expression analysis of ex vivo rabbit tendons.

## Data Availability

The dataset is stored at Mayo Clinic, Rochester, Minnesota. The raw data is available from the corresponding authors upon reasonable request.

## Abbreviations

Acan: Aggrecan
Acta: Actin Alpha
Actb: Actin Beta
Adamts4: ADAM Metallopeptidase With Thrombospondin Type 1 Motif 4
Alpl: Alkaline Phosphatase, Liver/Bone/Kidney
aMEM: Advanced Modification of Eagle’s Media
Col: Collagen
Dc: Decorin
ECM: Extracellular matrix
EtO: Ethylene oxide
FBS: Fetal bovine serum
FDR: False discovery rate
Fn: Fibronectin
Gapdh: Glyceraldehyde-3-Phosphate Dehydrogenase
Hapln1: Hyaluronan And Proteoglycan Link Protein 1
Hprt: Hypoxanthine Phosphoribosyltransferase
Ibsp: Integrin Binding Sialoprotein
Mmp: Matrix Metallopeptidase
N: Newton
PBS: Phosphate buffered saline
PMMA: Poly methyl methacrylate
RT-qPCR: Real time quantitative polymerase chain reaction
Ssp1: Secreted Phosphoprotein 1
Tcn: Tenascin C
