# Supplementary material for: A multi-chamber tissue culture device for load-dependent parallel evaluation of tendon explants
Source: BMC Musculoskelet Disord. 2019 Nov 18;20:549. doi: 10.1186/s12891-019-2896-2 (PMC6862789; doi:10.1186/s12891-019-2896-2)
Supplement: Supplementary file 1 — Additional file 1: RT-qPCR primers for gene expression analysis of ex vivo rabbit tendons. [file 12891_2019_2896_MOESM1_ESM.pdf]

Additional File 1. *qPCR primers for gene expression analysis of ex vivo rabbit tendons.*

Gene Name	GeneID	Forward primer sequences	Reverse primer sequences
Aggrecan	**Acan**	GAGGATGGCTTCCACCAGT	TGGGGTACCTGACAGTCTGA
Actin, Alpha1, Skeletal Muscle	**Acta1**	CACTGTGCCCATCTACGAGG	GTGGTCACGAAGGAGTAGCC
Actin Beta	**Actb**	GTGCGGGACATCAAGGAGAA	TTGCCGATGGTGATGACCTG
ADAM Metallopeptidase With Thrombospondin Type 1 Motif 4	**Adamts4**	AGTGTGCTGCCTACAACCAC	GGTGAGTTTGCATTGGTCTCG
Alkaline Phosphatase, Liver/Bone/Kidney	**Alpl**	CAAGCACTCCCACTTTGTC	ACCCAAGAGGTAGTCCACAG
Collagen Type I Alpha 1 Chain	**Col1a1**	GTAACAGCGGTGAACCTGG	CCTCGCTTTCCTTCCTCTCC
Collagen Type X Alpha 1 Chain	**Col10a1**	GGCTTCCCAGTGGCTGATAG	TTTTGCTCTCTCTGGGTGGC
Collagen Type II Alpha 1 Chain	**Col3a1**	GGACACAGAGGCTTCGAC	CCCTTTAATCCAGGAGCAC
Collagen Type V Alpha 1 Chain	**Col5a1**	CCGCCTACCAGAACGTCAC	ATGTAGGGGTTGTTGTCGTGG
Decorin	**Dcn**	GAGCTCCTGAACTTCCCGAC	AGGTCCTTGGGCACTTTGTC
Fibronectin 1	**Fn1**	GTTGATCAGCTGGGAAGCTC	ATCAATGGAAATTGGCTTGC
Glyceraldehyde-3-Phosphate Dehydrogenase	**Gapdh**	ACTTTGTGAAGCTCATTTCCTGGTA	GTGGTTTGAGGGCTCTTACTCCTT
Hyaluronan And Proteoglycan Link Protein 1	**Hapln1**	TTGAAGGGCTGGAAGACGAC	GCCTCGTGGAAGTTGAGGTT
Hypoxanthine Phosphoribosyltransferase	**Hprt**	ACGTCGAGGACTTGGAAAGG	GGGCTACAATGTGATGGCCT
Integrin Binding Sialoprotein	**Ibsp**	GTCTTTAAGTTCAGGCCACAG	ACTGCTCGGAACTGGAAAC
Matrix Metallopeptidase 1	**Mmp1**	TACCCCAAGGACATTCACAGC	CCTCAGAAACAGCAGCGTCAA
Matrix Metallopeptidase 3	**Mmp3**	CGGTTCCGCCTGTCTCAAG	CGCCAAAAGTGCCTGTCTT
Matrix Metallopeptidase 10	**Mmp10**	TCCACCAACCTCTCCTCGTA	ACAAAGCCGGATCGCACTTA
Matrix Metallopeptidase 13	**Mmp13**	ACAAACCACACTTGGGAGGG	ACTTGGGAATAGGCTTCCGC
Secreted Phosphoprotein 1	**Spp1**	GGAGACATGGAGGGAGATG	ATTCTCCGACTTGCTGTCC
Tenascin C	**Tnc**	AATCCTACCTGCCCTTGAACG	GGGATCGCCTTCCATTGCTT
